# More Attractive or More Interactive? The Effects of Multi-Leveled Emotional Design on Middle School Students’ Multimedia Learning

**DOI:** 10.3389/fpsyg.2019.03065

**Published:** 2020-01-22

**Authors:** Chenyu Shangguan, Zhen Wang, Shaoying Gong, Yawei Guo, Sheng Xu

**Affiliations:** ^1^School of Psychology, Central China Normal University, Wuhan, China; ^2^Key Laboratory of Adolescent Cyberpsychology and Behavior (CCNU), Ministry of Education, Wuhan, China; ^3^College of Education, University of Georgia, Athens, GA, United States

**Keywords:** multimedia learning, emotional design, visual design, behavioral design, middle school students

## Abstract

Previous studies on multimedia learning have provided shreds of evidence for the positive effect of visually attractive emotional design on college students’ emotion and learning outcomes. However, the effect may vary among middle school students. The aim of this study was to examine the impacts of visual and behavioral emotional design on the emotional, motivational and cognitive outcomes of middle school students. In Experiment 1, 50 participants (ages 13–15) were randomly assigned to one of two conditions: visual positive emotional design (colorful and anthropomorphic design) and visual neutral emotional design (achromatic and without anthropomorphic design). In Experiment 2, 173 participants (ages 13–16) were randomly assigned to one of four conditions created by the two factors: visual emotional design (positive vs. neutral) and behavioral emotional design (positive vs. neutral). The behavioral positive emotional design allows learners to interact with learning materials, whereas behavioral neutral emotional design only allows learners to watch learning video. Results showed that both visually attractive and behaviorally interactive design (visual positive emotion design and behavioral positive emotional design) had positive effect on learners’ positive emotions. Combining visual positive with behavioral positive emotional design could facilitate learning performance.

## Introduction

In educational settings, emotion has been regarded as a crucial factor influencing learning (Pekrun et al., 2002), and many studies have shown that positive emotions experienced by learners can promote learning (Pekrun et al., 2002; Pekrun, 2006). Thus, researchers and educators are beginning to focus on how to use educational technologies to enable learners to experience more positive emotions during the learning process. Indeed, research in the field of multimedia learning suggested that emotional design may be a promising way to improve learning via inducing learners’ positive emotions (Um et al., 2012; Mayer and Estrella, 2014; Plass et al., 2014). However, those studies focused more on visual attractive elements (e.g., color and anthropomorphism) and recruited only college students as participants (Uzun and Yıldırım, 2018). To fill these gaps in the literature on emotional design, the present study aimed to investigate the impact of multi-leveled (visual and behavioral) emotional design on multimedia learning of middle school students.

## Theoretical Framework

### Emotions and Multimedia Learning

Theoretical and empirical evidence has demonstrated that positive emotions could successfully facilitate learning in two different ways. First, the motivational benefits of emotions could promote learning (Moreno, 2007; Plass and Kalyuga, 2019). Specifically, positive emotions can enhance learners’ motivation, increase their engagement in learning tasks (Efklides et al., 2006), and further result in improved learning effects. Second, positive emotions could optimize the allocation of cognitive resources during learning (Plass and Kalyuga, 2019). To be specific, positive emotions may facilitate memory via increasing the amounts of cognitive resources and then lead to better learning performance.

However, a number of studies yielded mixed results. For example, some studies on the seductive detail effect indicated that the affective impact of decorative pictures could make learning more successful. In two experiments, Lenzner et al. (2013) compared the effects of decorative pictures with instructional pictures on physics learning, and results showed that decorative pictures induced better mood and instructional pictures were more beneficial for learning when combined with decorative pictures. Schneider et al. (2016) found that decorative pictures with positive emotional design successfully evoked positive emotions and in turn fostered retention and transfer performance. However, other studies demonstrated that decorative pictures negatively affected learning performance, although they successfully induced positive emotions (Rey, 2012, 2014; Gong et al., 2014). One possible explanation for this detrimental effect is that decorative pictures imposed extraneous cognitive load by forcing learners to spend their limited resources on processing materials that are unnecessary for mental model construction (Schnotz et al., 2009; Pekrun and Linnenbrink-Garcia, 2012; Park et al., 2015a; Plass and Kalyuga, 2019). As a consequence, in order to avoid the negative impact that decorative details may have on cognitive processes, the current study aimed to investigate the effects of positive emotions on cognitive processes and learning outcomes without adding additional learning materials.

### Emotional Design in Multimedia Learning

According to the cognitive-affective theory of learning with media (CATLM), emotional design not only helps to induce positive emotions but also affects learners’ emotional and motivational responses to learning materials and further promotes cognitive processing (Moreno, 2006). Emotional design was defined as the use of different design features with the goal of impacting learners’ emotions as a way to enhance learning (Park et al., 2015b). One thing should be noted that emotional design is the design of the intrinsic learning content and should not impose additional information on the learning topic (Navratil et al., 2018).

An important theory in emotional design was Emotional Design Model which proposes three levels of emotional design: visceral, behavioral and reflective level (Norman, 2004). The visceral level of emotional design is defined by how users perceive a product through their senses, such as the visual design, design layout and sound (Tractinsky et al., 2000; Norman, 2004). Visual design features such as color and graphics which can affect individuals’ perceived attractiveness and emotion are regarded as one of the most extensively used way of emotional design. The second level is the behavioral design which is usually associated with usability. Behavioral design is closely related with whether or not or how the product allows users to interact with the task (Kirschner et al., 2004; Sánchez-Franco et al., 2014). The reflective design is at the top of the model and is associated with the rationalization and intellectualization of a product or environment (Norman, 2004; Miller, 2011; Pengnate and Antonenko, 2013). The Emotional Design Model provides a general framework for designing and evaluating the learning materials in the multimedia learning environment (Norman, 2004).

#### Emotional Design Through Visual Design

Previous studies on emotional design in multimedia learning focused more on the visual level, and color and anthropomorphism have been identified as important visual design elements that could successfully evoke positive emotions (Wolfson and Case, 2000; Glocker et al., 2009). In particular, research indicated that warm colors could elicit greater and more positive emotional arousal than cold colors (Wolfson and Case, 2000), and baby-like anthropomorphism was positively associated with positive emotions (Glocker et al., 2009). However, recent research regarding the emotional and cognitive effects yielded mixed results. Some studies found the positive emotional design on visual elements could not only induce positive emotions but also facilitate cognitive outcomes including cognitive load and learning performance (Um et al., 2012; Mayer and Estrella, 2014; Plass et al., 2014, 2019; Gong et al., 2017a; Münchow et al., 2017; Brom et al., 2018; Schneider et al., 2018; Uzun and Yıldırım, 2018). For example, Plass et al. (2014) designed learning materials with warm color and baby-like shape design and found it could induce learners’ positive emotions, decrease perceived difficulty and promote learning performance. A recent meta-analysis (Brom et al., 2018) in the filed of multimedia learning reported that facial anthropomorphism and pleasant colors positively affected retention (*d* = 0.387), comprehension (*d* = 0.317), transfer (*d* = 0.327) and intrinsic motivation (*d* = 0.255), and reduced difficulty perception (*d* = −0.208), however, the two design elements had weaker effects on positive affect (*d* = 0.113). Plass et al. (2019) examined the emotional design of game characters, and results showed that warm colors and happy expressions were associated with adults’ happy emotion, whereas gray colors and sad expressions were associated with adults’ sad emotion. These findings were replicated in adolescent group. Some studies, however, failed to find the positive effects of visual emotional design on positive emotions (Münchow and Bannert, 2018; Stark et al., 2018a) or learning performance (Heidig et al., 2015; Park et al., 2015b; Brom et al., 2016). For instance, Brom et al. (2016) found that anthropomorphic and funny graphics within the animation learning had only a small positive impact on retention, and had no effects on transfer, state engagement and positive affect. Similarly, Park et al. (2015b) showed that anthropomorphism did not induce positive emotions and foster learning performance.

The following reasons may account for the inconsistency in these studies. First, visual emotional design elements vary in different studies. For example, some studies found only color design or only anthropomorphic design failed to induce positive emotions (Plass et al., 2014; Heidig et al., 2015) whereas the combination of color and anthropomorphism induced positive emotions (Um et al., 2012; Gong et al., 2017a), facilitated motivation (Um et al., 2012), cognitive load and learning performance (Mayer and Estrella, 2014). Second, previous studies have not distinguished between different levels of emotional design. For example, emotional text design which could be regarded as the design of learning content failed to affect learners’ positive emotion but led to better learning outcomes (Stark et al., 2018a) whereas visual emotional design combining both color and anthropomorphism induced positive emotions and facilitated learning (Um et al., 2012). Accordingly, the present study aimed to extend previous studies by exploring the emotional, motivational and cognitive effects of different levels of emotional design (visual design and behavioral design).

#### Emotional Design Through Behavioral Design

The Integrated Model of Multimedia Interactivity (INTERACT) describes learners’ actions in terms of behavioral, emotional, and cognitive activities and how these factors interact with each other to affect multimedia learning outcomes (Domagk et al., 2010). According to the INTERACT model, behavioral activities are a component of human-computer interaction, a prominent feature of which is learner control. Learner control, refers to the degree to which learners control different instructional characteristics in a course or program (Reeves, 1993), and learner control may be an important manipulation of behavioral emotion design. Existing research regarding the effects of the learner-paced instruction in multimedia learning or computer-based learning focused more on cognitive outcomes (e.g., cognitive resources, learning performance) and interactive behaviors (e.g., time-on-task, number of clicks) (Mayer and Chandler, 2001; Mayer et al., 2003; Hasler et al., 2007; Gerjets et al., 2009; Tabbers and de Koeijer, 2010; Gong et al., 2017b; Rey et al., 2019). For example, a recent meta-analysis demonstrated that learner-paced instruction had significant positive effect on cognitive load, retention and transfer performance, and increased learning time (Rey et al., 2019). Tabbers and de Koeijer (2010) found that learner-paced instruction not only improved transfer performance, but also increased learning time. However, studies in this area rarely investigated the emotional outcomes.

Research in the field of emotional design has also begun to pay attention to the effects of behavioral design. The behavioral level of emotional design is concerned with whether the use of a product is easy, pleasurable, and effective (Norman, 2004; Miller, 2011). Only a few studies have examined the effects of behavioral emotional design on learners’ learning process and performance. For instance, Heidig et al. (2015) found positive effects of behavioral design elements (usability of the learning environment) on emotional state. Miller (2011) designed a multimedia learning environment with visual (color design), behavioral (a horizontal bar-style graph of elapsed time for each phase of the performance tasks) and reflective design elements (an animated horizontal text ticker noting the current phase of each task). The results showed that such a design significantly decreased participants’ cognitive load and increased their satisfaction and task performance. However, Miller (2011) did not test the independent effects of the three levels of emotional design. Therefore, there is a lack of research on how to manipulate emotional design at the behavioral level and how behavioral emotional design affects learners’ emotional, motivational responses to the learning content.

### Problem Statement, Research Question and Hypotheses

Previous studies have been criticized for many reasons. First, most studies on emotional design were based on college students (Uzun and Yıldırım, 2018), therefore, whether the research results of visual design can be extended to other younger samples is unknown (Study 1). Second, existing research focused more on exploring visual emotional design elements and ignored other levels of design elements. Based on the Emotional Design Model, both visual and behavioral level were important aspects of emotional design (Norman, 2004) thus need to be explored (Study 2). Third, in most of the previous studies, the same learning topic and materials (“How does immunization work?”) were used. Whether the effects of emotional design have interdisciplinary stability needs further exploration (both Studies 1 and 2). To fill in these gaps, the present study aimed to explore the multi-leveled effects of emotional design (visual emotional design and behavioral emotional design) on positive emotions, cognitive load, motivation and learning outcomes of middle school students with a new learning topic.

Specifically, the research question of Study 1 was whether visual emotional design of multimedia learning materials induce middle school students’ positive emotions and lead to better cognitive and motivational outcomes. Three predictions were assumed in Study1: we hypothesized that compared with visual neutral design, visual positive emotional design can induce more positive emotions (H1), result in lower perceived task difficulty, higher mental effort, better learning performance (retention and transfer) (H2) and stronger motivation (H3). Study 2 explored the effect of visual emotional design again but more than that, the behavioral emotional design which was the second level of Emotional Design Model (Norman, 2004) was combined and explored. Overall, Study 2 aimed to test whether visual and behavioral emotional design can induce middle school students’ positive emotions, promote their cognitive and motivational outcomes in learning. We made the following hypotheses: We hypothesized that compared with neutral conditions, both positive visual design and positive behavioral design respectively, induce more positive emotions (H4), lower perceived task difficulty, higher mental effort, better learning outcomes (H5) and stronger motivation (H6).

## Study 1

The goal of Study 1 was to investigate whether visual emotional design can promote multimedia learning in middle school students. In line with previous studies (Um et al., 2012; Plass et al., 2014; Gong et al., 2017a), color and anthropomorphism were used as visual emotional design elements. The participants in Study 1 received the visual positive or visual neutral version of the learning material.

### Method

#### Participants and Design

G^∗^power was used to estimate the sample size (Faul et al., 2007) with the effect size of 0.8 and power of 0.8 (see Cohen, 1988). We then aimed for a sample size of minimum 21 participants for each group. In this study, participants were 50 middle school students (29 male and 21 female; age: *M* = 13.90, *SD* = 0.68) recruited from a middle school. They had normal or corrected-to-normal vision. Participants were randomly assigned to one of two conditions: visual positive emotional design (VP, *n* = 27) or visual neutral emotional design (VN, *n* = 23). The study protocol was approved by the Ethical Committee of the School of Psychology at Central China Normal University. Permission for the study was obtained from school authorities and principles. Written informed consent was obtained prior to starting the experiment from both the school and parents. Informed consents of Study 2 were consistent with Study1.

#### Materials and Measures

##### Design of learning materials

**FIGURE 1:**
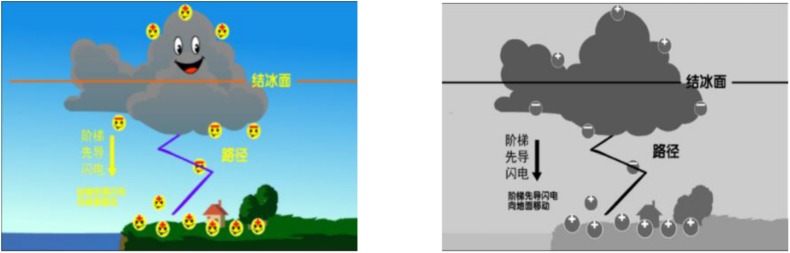
Screenshots of multimedia learning materials from Study1: on left, visual positive emotional design (VP); on right, visual neutral emotional design (VN).

Multimedia learning material with the topic of “The Formation of Lightning” was presented in computers (Mayer, 2009; Gong et al., 2017a). The learning material was a Flash animation which lasted 152 s and was program-controlled. Two different versions of visual emotional design were used in the present study. Visual positive emotional design (VP) used colorful and anthropomorphic elements. The anthropomorphic design aimed to make the elements in the learning material (e.g., sun, cloud, electric charge) more expressively anthropomorphic. The color design didn’t limit to warm colors (e.g., Um et al., 2012), but applied natural colors as similar to the color of real elements. Visual neutral emotional (VN) materials were developed with achromatic design and without anthropomorphism (see Figure 1).

##### Emotional state

To check the effectiveness of the mood induction, a Positive Emotion Self-Report Inventory was used (e.g., Gross and Levenson, 1995). According to previous studies (e.g., Chaffar and Frasson, 2004; Gong et al., 2017a), six items that related to positive emotions were used in this study (happy, excited, content, active, interested and relaxed). Respondents indicated the degree to which they experienced six positive emotions, using a 9-point Likert scale ranging from 1 (not at all) to 9 (very much). The total score for each participant was obtained by averaging the scores for the 6 responses. This scale had high internal consistency in this study (coefficient α = 0.92).

##### Motivation

A 7-point self-report instrument (Isen and Reeve, 2005) that has been widely used in previous studies (Um et al., 2012; Gong et al., 2017a) was used to measure learners’ motivation. Participants were required to rate their motivation of the learning experience (e.g., “It piqued my curiosity,” 1 = strongly disagree, 7 = strongly agree). The final score was obtained by averaging the scores for all the responses (coefficient α = 0.92).

##### Cognitive load

One item was used to measure learners’ mental effort (“How much mental effort did you pay out in learning the materials?,” 1 = none, 9 = extremely much) (Paas, 1992). One item was to measure the perception of task difficulty (“How easy or difficult was the material to understand?”, 1 = extremely easy, 9 = extremely hard) (Kalyuga et al., 2000). They measured different structures and were successfully used in previous studies (e.g., Um et al., 2012).

##### Retention and transfer tests

To measure learners’ memory and understanding of key concepts, the retention test was applied with 9 multiple-choice questions (e.g., “How does lightning form?”). Participants got one point for each correct answer, with a maximum score of 9 points for the retention test. Transfer test was used to measure the transfer of knowledge with three subjective questions (e.g., “Why are there sometimes clouds but no lightning in the sky?”). Answers were assigned 1 point for each key point included, with a maximum of 10 points assigned to each answer. The total possible score on the transfer test was 30 points. Two raters rated the transfer test and the inter-rater reliability is 0.95.

##### Control measures

Prior knowledge was assessed using a 4-item self-report checklist, 5 multiple-choice questions and a subjective question to test their level of knowledge about the learning topic “the formation of lightning.” The four self-report items (e.g., “I know the formation of a cloud”) ranged from 0 (I have no idea at all) to 4 (I totally understand). Five multiple-choice questions (e.g., How does a cloud form? A. Air flow rises, B. Temperature falls, C. Water vapor condenses into water droplets, D. Do not know) were assigned 1 point for each correct answer. The subjective question (“Please write down everything you know about the formation of lightning”) had a maximum score of 10. One point was awarded for each key point. The learner’s total score on the prior knowledge test was obtained by adding points from all items, ranging from 0 to 31 points.

Learning interest. Interest in physics was controlled using a 9-point Likert-type question (“How about your interest in physics?”, 1 = not interested at all, 9 = extremely interested). Interest in the learning topic was also measured using a 9-point Likert-type question (“How about your interest in the topic ‘the formation of lightning’ that you are going to learn?”, 1 = not interested at all, 9 = extremely interested).

#### Procedure

**FIGURE 2:**
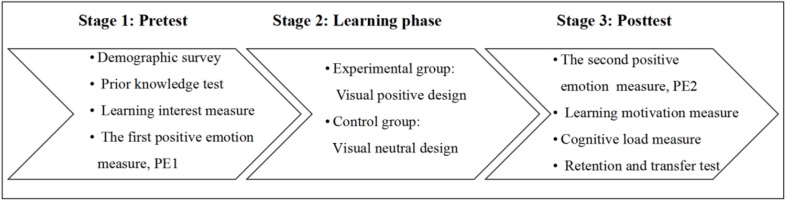
Procedure of the study.

The learning materials were presented using a computer program. Each participant was individually tested in the computer room and the whole process lasted for about 45 min. The procedure can be seen in Figure 2.

First, the experimenter briefly introduced the procedure to participants. Next, participants were asked to complete a demographic survey, the prior knowledge questionnaire, learning interest questionnaire, and positive emotion questionnaire (the first positive emotion measure, PE1). Thereafter, the participants were presented the materials using either visual positive or visual neutral design. Soon after learning, participants completed the positive emotion measure for the second time (PE2), and then responded to items about cognitive load, the motivation questionnaire, learning performance (retention and transfer tasks). The transfer test was completed in paper-and-pencil measures.

#### Analyses

We firstly performed independent samples *t* test to check for differences between conditions regarding the prior knowledge, PE1 and learning interest. Secondly, to test H1, we conducted repeated measures analysis of variance (RM-ANCOVA) with visual emotional design as the between-subjects factor, positive emotion measures (PE1 and PE2) as the within-subjects factor, and interest in physics and topic as covariates. Moreover, we tested H2 and H3 using ANCOVAs with visual emotional design as the independent variable, and interest in physics and topic as covariates.

### Results

Table 1 presents the descriptive statistics for all dependent and control variables. Results of independent samples *t* tests showed no group difference on the first positive emotion score, *t* (48) = 0.72, *p* = 0.48, and prior knowledge, *t* (48) = 0.93, *p* = 0.36. There were group differences on interest in physics, *t* (48) = 2.58, *p* = 0.01, *d* = 0.73, and interest in topic, *t* (48) = 2.24, *p* = 0.03, *d* = 0.63. Interest in physics and topic were treated as control variables in the following analyses.

**TABLE 1:** Means and standard deviations of all variables for the two groups.

	VP (*n* = 27)	VN (*n* = 23)
	*M* (*SD*)	*M* (*SD*)
Prior knowledge	16.85 (3.15)	17.78 (3.94)
Positive emotion (1)	5.43 (1.79)	5.80 (1.80)
Positive emotion (2)	5.83 (1.99)	5.05 (1.72)
Retention	3.78 (1.70)	3.52 (1.93)
Transfer	4.52 (5.16)	4.00 (4.79)
Perceived difficulty	6.11 (1.81)	5.26 (1.29)
Mental effort	6.11 (1.81)	5.35 (1.77)
Motivation	5.19 (1.14)	5.06 (1.09)
Interest in physics	5.89 (1.63)	7.09 (1.65)
Interest in topic	6.26 (1.83)	7.39 (1.73)

*VP-visual positive emotional design; VN-visual neutral emotional design.*

#### Positive Emotions

**FIGURE 3:**
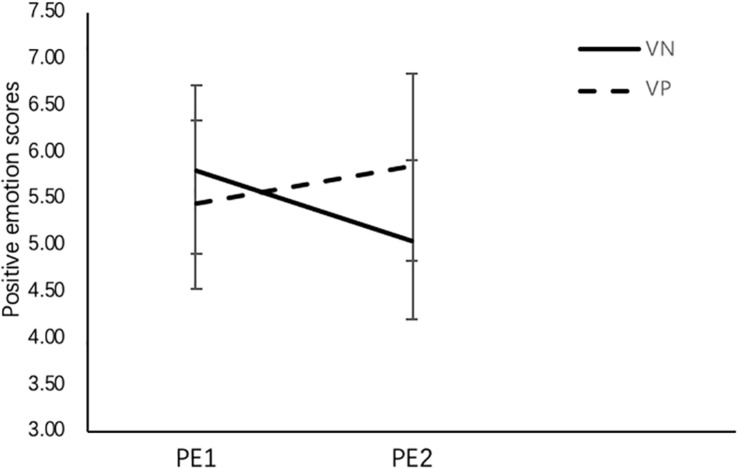
Positive emotions by condition before learning (PE1) and after learning (PE2). PE1, first positive emotion measure; PE2, second positive emotion measure; VN, visual neutral emotional design; VP, visual positive emotional design. Standard deviations were showed in the figure.

Regarding the effects of visual positive emotional design on positive emotions, the results of RM-ANCOVA revealed that there were no main effects of visual emotional design, *F* (1,46) = 3.43, *p* = 0.07, and positive emotion measures, *F* (1,46) = 0.14, *p* = 0.72, but there was a significant interaction effect between visual emotional design and positive emotion measure, *F* (1,46) = 6.13, *p* = 0.02, η*^2^_*p*_* = 0.12 (see Figure 3). Follow-up analysis revealed that on PE2, positive emotion in the VP group was significantly higher than in the VN group, *F* (1,46) = 7.61, *p* = 0.01, η*^2^_*p*_* = 0.14.

#### Motivation

Concerning learners’ motivation as dependent variable, the results of ANCOVA showed no main effect of visual emotional design, *F* (1,46) = 2.70, *p* = 0.11.

#### Cognitive Load

With regard to the effects of visual emotional design on learners’ cognitive processes, the results of ANCOVAs revealed a main effect of visual emotional design on perceived difficulty, *F* (1,46) = 7.04, *p* = 0.01, η*^2^_*p*_* = 0.13, with the visual positive emotional design group perceiving significantly higher difficulty than the visual neutral group. Results also showed a main effect of visual emotional design on mental effort, *F* (1,46) = 4.65, *p* = 0.04, η*^2^_*p*_* = 0.09, with the visual positive emotional design lead to higher mental effort than the visual neutral design.

#### Learning Performance

Regarding retention and transfer scores as dependent variables, the results of ANCOVAs revealed no main effects of visual emotion design on retention, *F* (1,46) = 0.42, *p* = 0.52, or transfer, *F* (1,46) = 0.25, *p* = 0.62.

### Brief Discussion

The purpose of Study 1 was to test emotional, motivational and cognitive effects of visual emotional design on middle school students. Results indicated that visual positive emotional design could successfully induce positive emotions, increase mental effort and subjective task difficulty. These findings put new insights on how visual emotional design affects learners’ learning processes and learning outcomes.

Firstly, results showed that compared with the visual neutral emotional design group, the visual positive emotional design group experienced significantly more positive emotions (support for **H1**). This result indicated that visual positive emotional design, which combined color and anthropomorphic elements, helped induce and maintain middle schoolers’ positive emotions during learning. By replicating earlier results among college learners (Um et al., 2012; Mayer and Estrella, 2014; Gong et al., 2017a), we added evidence in support of the effectiveness of visual positive emotional design in the middle school population.

Concerning cognitive outcomes, the results showed that visual positive emotional design increased learners’ mental effort and subjective task difficulty (partial support for **H2**). Learners maintained more positive emotions in the VP condition, which in turn, helped them focus more attention on the details of the learning materials. As a consequence, they reported more mental effort in learning (Paas and Sweller, 2014). Unexpectedly, our results also indicated that learners in the visual positive emotional design condition may experience a higher level of task difficulty during learning, as not only do they need to mentally process learning content with emotional design but also allocate cognitive resources for emotional regulation. In addition, our data did not support the hypothesis that visual positive emotional design could facilitate learning outcomes (either retention or transfer). One reason may be that compared with college students, middle school students may have more difficulty in applying knowledge to solve practical problems. Closely checking the learners’ transfer scores (*M* = 4.28, *SD* = 4.95, with a maximum score of 30), it appears the transfer test may have had a “floor effect”.

Regarding the motivational outcome, no positive effect of visual emotional design on motivation was found in the present study (a lack support of **H3**), which was not consistent with some of the previous studies (e.g., Gong et al., 2017a; Navratil et al., 2018). It could be interpreted as the motivation decreasing function of the learning material itself (Knörzer et al., 2016; Stark et al., 2018a). In addition, the present study recruited middle school students as participants who were less familiar with the learning topic than college students because of less physics knowledge and less life experience, therefore, it may be much harder to maintain the motivation of middle school students during the whole learning process.

Findings from Study 1 indicated that middle school students could have difficulty in applying knowledge they learned into practice to solve real problems. Based on these findings, we wondered whether giving them the opportunity to control their learning process would increase their positive emotion, promote their deep understanding of learning materials, and facilitate transfer. After partially replicating findings from previous studies on college students (Um et al., 2012; Plass et al., 2014; Gong et al., 2017a), we next introduced another level of emotional design, namely behavioral emotional design, to the model. That is, we tested the effects of both the visual and behavioral levels of emotional design on the multimedia learning of middle school students.

## Study 2

Study 2 took both visual and behavioral level of emotional design into consideration and aimed to test whether visual and behavioral emotional design can induce middle school students’ positive emotions and promote their multimedia learning.

### Method

#### Participants and Design

G^∗^power was used to estimate the sample size (Faul et al., 2007) with the effect size of 0.25 and power of 0.8 (see Cohen, 1988). We then aimed for a total sample size of 179 participants. In this study, participants were 173 middle school students (79 male and 94 female; age: *M* = 14.75, *SD* = 0.79) recruited from a middle school. They had normal or corrected-to-normal vision. The study used a 2 × 2 between-subjects design: the first factor was visual emotional design (positive vs. neutral) and the second factor was behavioral emotional design (positive vs. neutral). Participants were randomly assigned to one of the four conditions:

(a) visual positive and behavioral positive emotional design condition (VPBP, *n* = 45);
(b) visual positive and behavioral neutral emotional design condition (VPBN, *n* = 43);
(c) visual neutral and behavioral positive emotional design condition (VNBP, *n* = 45); and
(d) visual neutral and behavioral neutral emotional design condition (VNBN, *n* = 40).

The visual level of emotional design was the same as in Study 1, combining color and anthropomorphism to design the learning material, while the behavioral level of emotional design applied learner control during learning according to the research of Stark et al. (2018b). The participants in behavioral positive emotional design condition could self-control the whole learning progress by fast-forwarding, rewinding or repeating the learning video. The behavioral neutral conditions were program-controlled.

#### Materials

##### Design of learning materials

The learning material in Study 2 was the same as in Study 1.

##### Measures and procedures

The measures were the same as those used in Study 1. The procedures were the same except that behavioral positive emotional design (BP) group could interact with the learning environment (self-control their learning progress).

##### Analyses

First of all, one-way ANOVA was used to explore the differences between conditions regarding the PE1 and learning interest, and the independent-samples *t* test was applied to checked for the manipulation of behavioral emotional design. Then, we conducted RM-ANOVA with four experimental conditions as the between-subjects factor, positive emotion measures (PE1 and PE2) as the within-subjects factor for a manipulation check. Meanwhile, paired-sample *t* tests were conducted to investigate the changes of positive emotional scores in four experimental conditions during the learning process. Thereafter, we performed 2 × 2 ANOVA with visual emotional design and behavioral emotional design as between-subject factors, positive emotions scores, cognitive load, learning performance and learning motivation as dependent variables to test H4, H5, and H6.

### Results

Table 2 presents the descriptive statistics for all dependent and control variables. A one-way ANOVA showed no difference in PE1 between the four groups, *F* (3,169) = 1.75, *p* = 0.16. Concerning control variables, results of one-way ANOVAs showed no group differences in interest in physics, *F* (3,169) = 1.00, *p* = 0.39, interest in topic, *F* (3,169) = 0.13, *p* = 0.94, and prior knowledge, *F* (3,169) = 1.56, *p* = 0.20. We then checked the manipulation of behavioral emotional design. An independent-samples *t* test revealed that the learning time of learners in behavioral positive emotional design group condition (*M* = 344.11, *SD* = 214.36) was significantly longer than that in behavioral neutral design condition (*M* = 165.14, *SD* = 20.05), *t* (171) = 7.57, *p* < 0.001, *d* = 1.20, which indicated that learners in the positive behavioral emotional design group did interact with the learning material.

**TABLE 2:** Means and standard deviations of all variables for the four groups.

	VPBP	VPBN	VNBP	VNBN
	*n* = 45	*n* = 43	*n* = 45	*n* = 40
	*M* (*SD*)	*M* (*SD*)	*M* (*SD*)	*M* (*SD*)
Prior knowledge	14.89 (3.98)	14.14 (2.95)	16.80 (2.87)	13.40 (3.39)
Positive emotion (1)	5.56 (1.55)	5.75 (1.48)	6.14 (1.49)	6.17 (1.46)
Positive emotion (2)	5.87 (1.34)	5.33 (1.82)	5.64 (1.30)	5.00 (1.85)
Retention	5.89 (1.57)	5.60 (2.00)	5.47 (1.84)	5.18 (2.04)
Transfer	7.60 (6.06)	7.09 (5.18)	5.89 (4.22)	5.10 (3.87)
Perceived difficulty	5.24 (1.21)	5.23 (1.74)	5.38 (1.47)	5.48 (1.95)
Mental effort	6.42 (1.67)	5.84 (1.63)	6.00 (1.60)	6.05 (1.50)
Motivation	4.88 (1.02)	4.86 (1.14)	4.84 (1.02)	4.84 (1.34)
Interest in physics	6.36 (1.30)	5.79 (1.87)	5.84 (1.72)	6.00 (1.85)
Interest in topic	6.38 (1.67)	6.26 (1.71)	6.16 (2.01)	6.35 (2.12)
Learning time	318.71 (185.45)	161.40 (10.34)	369.51 (239.23)	169.18 (26.42)

*VPBP-visual positive and behavior positive design; VPBN-visual positive and behavior neutral design; VNBP-visual neutral and behavior positive design; VNBN-visual neutral and behavior neutral design.*

#### Positive Emotions

**FIGURE 4:**
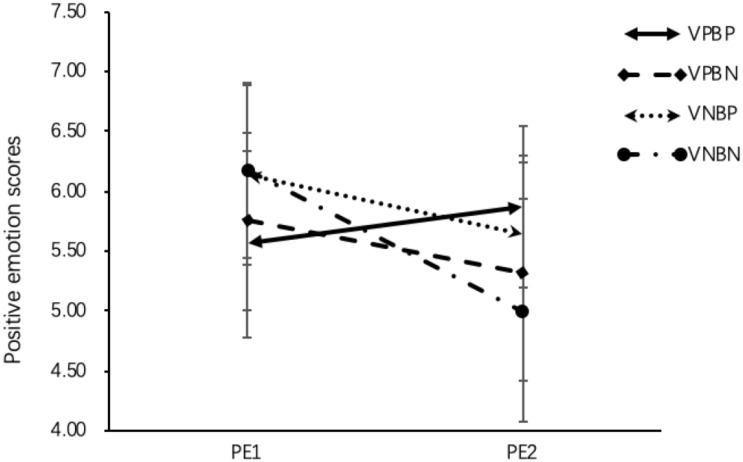
Positive emotions by condition before learning (PE1) and after learning (PE2) in Study 2. PE1, first positive emotion measure; PE2, second positive emotion measure; VPBP, visual positive and behavior positive design; VPBN, visual positive and behavior neutral design; VNBP, visual neutral and behavior positive design; VNBN, visual neutral and behavior neutral design. Standard deviations were showed in the figure.

Firstly, the results of RM-ANOVA with four experimental condition as between-subject factor and positive emotion measures (PE1 and PE2) as repeated measures indicated no main effect of condition, *F* (3,169) = 0.65, *p* = 0.58. There was a main effect of positive emotion measures, *F* (3,169) = 12.31, *p* < 0.001, η*^2^_*p*_* = 0.07 and the interaction effect was also significant, *F* (3,169) = 5.51, *p* < 0.001, η*^2^_*p*_* = 0.09. Regarding whether the positive emotion scores (from PE1 to PE2) changed significantly over the learning process, the results of paired-sample *t* tests revealed that (see Figure 4): for the VPBP group, there was no significant change from PE1 to PE2, *t* (45) = −1.22, *p* = 0.23. As for VNBP and VNBN group, the scores in positive emotion reduced significantly from PE1 to PE2, *t* (45) = 2.62, *p* = 0.01, *d* = 0.36; *t* (40) = 3.41, *p* < 0.001, *d* = 0.70. For the VPBN group, positive emotions decreased marginally significantly from PE1 to PE2, *t* (43) = 1.86, *p* = 0.07, *d* = 0.25.

Concerning the effects of visual and behavioral emotional design on positive emotions, the results of ANOVA showed no main effect of visual emotional design, *F* (1,169) = 1.30, *p* = 0.26, but there was a main effect of behavioral emotional design, *F* (1,169) = 5.98, *p* = 0.02,η*^2^_*p*_* = 0.03. Behavioral positive emotional design induced more positive emotions than behavioral neutral emotional design. No interaction effect was observed, *F* (1,169) = 0.04, *p* = 0.85.

#### Cognitive Outcomes and Motivation Outcomes

We tested the effects of visual and behavioral emotional design on learning outcomes using ANOVAs, with retention and transfer test as dependent measures. The analyses revealed no main effect of visual emotional design on retention, *F*(1,169) = 2.25, *p* = 0.14, but a significant main effect on transfer, *F*(1,169) = 6.13, *p* = 0.01, η*^2^_*p*_* = 0.04. Learners in the visual positive emotional design condition scored significantly higher on the transfer test than those in the visual neutral emotional group. There was no main effect of behavioral emotional design on retention, *F*(1,169) = 1.03, *p* = 0.31, or on transfer, *F*(1,169) = 0.75, *p* = 0.39. There was no interaction effect on retention, *F*(1,169) = 0.00, *p* = 0.99, or transfer, *F*(1,169) = 0.04, *p* = 0.85.

Concerning learners’ perceived difficulty, mental effort and motivation as dependent measures, the results of ANOVAs showed that the main effects and the interaction effects were all not significant, *F*_*s*_ < 0.88, *p_*s*_* > 0.05.

### Brief Discussion

Based on Study 1 and the emotional design model (Norman, 2004), Study 2 tested the effects of visual and behavioral levels of emotional design on the multimedia learning of middle school students. The results showed that behavioral emotional design significantly induced positive emotions of learners (in partial support of **H4**), which suggests that interacting with learning environment can facilitate positive emotions in middle school students. The findings provide evidence for the INTERACT model demonstrating that interactive behavior in learning environment can induce learners’ positive emotions (Moreno and Mayer, 2007; Domagk et al., 2010). Especially when learners have more control over the learning process, they may experience more positive emotions. The results also revealed that learners in the VPBP were more likely to maintain positive emotions than the other three groups (in partial support of **H4**). This result suggests that the combination of different levels of emotional positive design may be more conducive to maintaining the learners’ positive emotions than a single level. Findings concerning the benefits of behavioral design is partly consistent with previous studies based on college students (Um et al., 2012; Plass et al., 2014; Gong et al., 2017a).

In regard to the cognitive process outcomes (mental effort and perceived task difficulty) and learning outcomes (retention and transfer), the results only showed that transfer scores were significantly higher in the visual positive emotional design group than in the visual neutral emotional design group (in partial support of **H5**). Beyond our expectation, combining visual and behavioral level of emotional design only facilitated learners’ positive emotions but had no significant positive effect on learners’ cognitive process outcomes and learning performance. A possible explanation may be that when combining visual and behavioral positive emotional design elements at the same time, the visually attractive design distracted learners’ cognitive resources and the behavioral interactive design allowed learners to interact more with the learning material, thus less attention was paid to perceive their cognitive process. Additionally, an alternative explanation is that the indicator of the cognitive process used in the present study was self-reported cognitive load by learners, which may not have been sufficiently sensitive to changes in perceived levels of cognitive load. Indeed, how to accurately measure the cognitive load experienced by learners remains a challenge (Korbach et al., 2017). Future research should consider including other cognitive outcome variables, such as cognitive engagement. Moreover, consistent with some studies indicating that although there was no improvement in cognitive processing, but the learning performance was enhanced (e.g., Mayer and Estrella, 2014). We observed a positive effect of visual positive emotional design on learners’ transfer, especially in the behavioral positive emotional design condition. As in the VPBP condition, more positive emotions were maintained during the whole learning process, thus positive emotion will facilitate learning (Pekrun et al., 2006; Knörzer et al., 2016). Based on the discussion above, we suggest combining both visual positive with behavioral positive emotional design could facilitate learning performance.

Regarding the motivation, no main effect or interaction effect of visual and behavioral emotional design was found (a lack of support for H6). The result was consistent with the results in motivation in Study 1 and it could be inferred that for middle school students, more exploration needed to be done to help them induce and maintain motivation during learning.

Overall, the results in Study 2 were not replicated the findings on college students (Um et al., 2012; Gong et al., 2017a) since the participants in our study were middle school students who likely had less experience with multimedia learning. In addition, based on the results of descriptive statistics on perceived task difficulty variables, the task difficulty of the present study is moderate. However, recent research suggested that learner control is more effective in enhancing learners’ performance on complex tasks (Gong et al., 2017a). Thus, task difficulty may influence the benefits of behavioral emotional design on cognitive outcomes. Moreover, we can infer that the emotional design effect from college students may not be directly translated to a younger audience (Hubisz, 2000; Mctigue, 2009). Future studies need to further explore the effects of emotional design on different populations.

## General Discussion

In order to reveal the role of emotional design in multimedia learning more clearly and systematically, this study examined the impact of different levels of emotional design on the learning process and learning outcomes of middle school students. Results indicated that both attractive and interactive design (the visual positive emotion design and the behavioral positive emotional design) have positive effects on learners’ positive emotions. Combining visual positive with behavioral positive emotional design could facilitate learning performance. This study is the first attempt to investigate the effect of multi-leveled emotional design on middle school students’ emotional, motivational and cognitive outcomes.

### The Effects of Multi-Leveled Emotional Design on Learning

Concerning the positive emotion outcomes, the results of two studies in middle school samples provide evidence that visual emotional design (colorful and anthropomorphic design) can help maintain positive emotions produced before learning, and behavioral positive emotional design (behavioral interact with learning material) can induce and sustain positive emotions. The present research found consistent results with previous studies (Plass et al., 2014; Gong et al., 2017a; Uzun and Yıldırım, 2018) showing that both visual and behavioral positive emotional design can help maintain positive emotions during learning. The specific result showing that behavioral emotional design induced positive emotions was consistent with the proposals of the control-value theory of academic emotions (Pekrun et al., 2002; Pekrun, 2006). When learners feel more control over their learning process, they will experience more positive emotions. Beyond previous research, the present study tested the effects of two levels of emotional design on emotions with a younger sample.

Concerning the cognitive outcomes, the results of the two experiments depict a complex picture of how visual and behavioral level of emotional design affected learners’ cognitive process outcomes and learning performance. The results of Study 1 found that visual positive emotional design increased learners’ cognitive process outcomes (increased mental effort) but had no positive effect on learning outcomes whereas the results of Study 2 revealed no positive effect of visual positive emotional design on cognitive process outcome, but visual positive emotional design facilitated learning outcomes. The learning situation may provide a possible explanation for this inconsistency. Study 2 partly allowed participants to interact with the learning environment, which might be more interesting for middle school students. Thus, they devoted more attention to the learning materials and less attention to perceive their cognitive processes. In addition, an important factor that could influence learning processing and performance was learners’ individual difference, especially their prior knowledge (Kalyuga et al., 1998; Kalyuga, 2009). One thing needs to be point out is that the prior knowledge of learners in Study 2 was lower than that in Study 1, which might affect learning processes and outcomes (Korbach et al., 2017).

Overall, our findings partly replicated but not all of the results based on college students. A potential reason is that most of the participants in the current research were in ninth grade and under great pressure due to the high school entrance exam; their emotional experience and emotional perception are also less developed than college students (Valsiner and Connolly, 2003). And a meta-analysis revealed that learners’ age is one of the important moderators influencing learning effect (Schroeder et al., 2013). This suggests the need to exercise caution when applying multimedia learning principles (Mayer, 2005, 2009) in a younger audience. Another reason for the inconsistent results may be the use of different emotion induction procedures or different multimedia materials and instructions across studies. Mood-induction in some studies combined both external mood induction (e.g., inducing mood by video before learning) and emotional design induction (Um et al., 2012; Plass et al., 2014).

### Implications

The current study provides positive evidence that designing the multimedia learning materials in multi-leveled ways improves learners’ positive emotions, especially by behavioral positive emotional design. In addition, the positive effect of positive emotional design on both cognitive processing (Study 1) and learning performance (Study 2) was observed. Concerning the theoretical contributions of the present study, the results are consistent with the predictions of CATLM which suggests that adding affective factors can be beneficial for learning. Based on CATLM, emotional design features cause learners to devote more cognitive resource (e.g., mental effort) to learning process and this will in turn facilitate learning performance. In addition, the present study is also consistent with the INTERACT model (Domagk et al., 2010). That is, learners’ behavioral activity in learning environment demonstrating what learners physically do to interact with learning system can affect learners’ emotion and also cognitive activity. Meanwhile, the findings of the present study could provide practical suggestions for the design in multimedia learning environment. Specifically, when instructional designers applied emotional design principles in learning, it is important to comprehensively take the design elements (e.g., visually attractive and behaviorally interactive emotional design) and learners’ characteristics (e.g., age) into consideration.

### Limitations and Future Research

However, there were still some limitations of our study. First, the learning content in the present study was science principles. However, previous research indicated that learning content was considered as an important factor affecting multimedia learning (Schroeder et al., 2013). Thus, future research should apply different discipline knowledge to examine the effects of emotional design. Second, although we explored the effects of behavioral emotional design with pace-control, it needs to be pointed out that pace-control is a relatively limited approach reflecting the behavioral emotional design dimension. Future research needs to investigate what kind of interactive behavior design can induce learners’ positive emotions and promote learning. Third, several one-item scales were used in the present study, which makes it difficult to interpret the reliability. Future studies should apply more effective measurements of learning processes and outcomes. Fourth, the final number of participants in each condition in both Study 1 and Study 2 were not strictly the same, which might cause some errors. Future research should carefully choose the number of participants before the study as well as ensured the final number in each experimental condition to be consistent to prevent possible experimental deviation. In addition, self-reported measures were used in the current study. As participants may not be very sensitive to their real-time emotional state and cognitive processes, future research should use more direct measures of emotion and cognition in relation to design issues in multimedia learning. Finally, as a recent meta-analysis study revealed that the processing of multimedia materials could be different between male and female (Castroalonso et al., 2019), the gender variable could be included in future research on instructional visualizations in multimedia learning.

## Data Availability Statement

The datasets generated for this study are available on request to the corresponding author.

## Ethics Statement

The studies involving human participants were reviewed and approved by Ethical Committee of the School of Psychology at Central China Normal University. Written informed consent to participate in this study was provided by the participants’ legal guardian/next of kin.

## Author Contributions

CS: conceptualization, acquisition, collecting, analysis, inter- pretation, and drafting. ZW: interpretation, writing, and revising the work. SG: conceptualization, interpretation, revising the work, supervision, and validation. YG: revising the draft. SX: designing the learning system.

## Conflict of Interest

The authors declare that the research was conducted in the absence of any commercial or financial relationships that could be construed as a potential conflict of interest.
